# Graft-Versus-Host Disease in Allogeneic Hematopoietic Stem Cell Transplant: SARS-CoV-2 Vaccine as a Potential Trigger

**DOI:** 10.7759/cureus.25738

**Published:** 2022-06-07

**Authors:** Amin Azem, Zainub Ajmal, Mihir Raval

**Affiliations:** 1 Internal Medicine, Albany Medical Center, Albany, USA; 2 Hematology and Oncology, Albany Medical Center, Albany, USA; 3 Hematology and Oncology, New York Oncology and Hematology, Albany, USA

**Keywords:** acute lymphoblastic leukemia (all), acute myeloid leukemia (aml), acute graft vs host disease, allogeneic bone marrow transplant, sars-cov-2 vaccine

## Abstract

Allogeneic hematopoietic stem cell transplant (AHSCT) recipients are at a risk of developing immune-mediated tissue damage from activation of the donor’s immunocompetent T cells by the recipient’s normally expressed antigens, a phenomenon called graft-versus-host disease (GVHD). With the emergence of the novel severe acute respiratory syndrome coronavirus 2 (SARS-CoV-2), new vaccines have been developed to prevent morbidity and mortality, including the highly vulnerable hematologic malignancy patients after undergoing AHSCT. The early pathophysiologic events in GVHD include priming the donor T cells with molecules that are endogenous or pathogenic. In this case series, we present two cases of AHSCT recipients in which the SARS-CoV-2 vaccination series proceeded the development of GVHD manifesting with oral mucosal symptoms and derangement in the liver function tests. Our experience raises the question if any of the vaccine components serve as a molecular trigger for GVHD, making the current SARS-CoV-2 vaccines a risk factor for activating the immune system and developing GVHD.

## Introduction

Determining the eligibility of allogeneic hematopoietic stem cell transplant (AHSCT) in patients with certain hematological diseases such as acute leukemia is essential upon initial evaluation as it is part of standard therapy to maintain remission in these patients. One of the well described and severe complications of AHSCT is graft-versus-host disease (GVHD) in which the donor’s immunocompetent T cells recognize the recipient’s antigens as non-self, resulting in immune activation of these T cells attacking the recipient’s normal cells [1]. The three most common organs involved in acute GVHD are the skin, gastrointestinal tract, and liver [2]. The risk factors for developing GVHD include increasing age, the degree of HLA mismatch, gender mismatch, and the source of stem cell with risk being higher with peripheral blood stem cell transplantation [3]. The risk is also lower with reduced-intensity regimens compared with myeloablative regimens [4]. The diagnosis of GVHD is based on clinical, laboratory, and histopathologic findings, and the severity of GVHD is graded from 1 to 4 (mild, moderate, severe, and very severe). It is estimated that 30% to 50% patients have acute GVHD (grades 1-4) and 14% have severe acute GVHD (grades 3-4) [1].

Infections in AHSCT patients may be a trigger for an immune response culminating with GVHD [1]. AHSCT patients receive vaccinations against common vaccine preventable diseases (e.g., tetanus, shingles, influenza, hepatitis, measles, mumps, and rubella). The Center for Disease Control and Prevention (CDC) recommends administration of these vaccines when CD4 count is ≥ 200. For a vaccine to mount a response that is clinically relevant, the immune system has been reconstituted with adequate numbers and function of B and T lymphocytes to a degree that allows formation of antigen-specific responses [5]. Thus, the earliest time frame to start administering most vaccines is around six months after transplant. Some patients may already have active GVHD by the time they are eligible for vaccinations, and the current recommendations are to delay administering vaccines rather than holding off entirely in these cases [6]. Patients with hematological malignancies have a higher risk of developing severe respiratory illness and mortality after infection with severe acute respiratory syndrome coronavirus-2 (SARS-CoV-2) [7]. The use of vaccines against SARS-CoV-2 for AHSCT recipients is currently being studied, and there are emerging data regarding the side effect profile in this group of patients. Here we present two cases of AHSCT recipients developing GVHD after receiving SARS-CoV-2 vaccinations and how they were subsequently treated.

## Case presentation

Case 1

The first case is of a 58-year-old male who initially presented in 2018 with generalized weakness and workup revealed leukocytosis of 144,000, as he was complaining of general weakness. His peripheral flow cytometry and bone marrow biopsy in December 2018 confirmed the diagnosis of acute myeloid leukemia (AML). Cytogenetic testing did not reveal any mutations. Molecular analysis was positive for FLT3, NPM1, and TET2 mutations. He was started induction therapy with standard 7+3 plus midostaurin and after achieving remission. He received three cycles of consolidation high-dose cytarabine plus midostaurin. He subsequently underwent AHSCT in May 2019. His transplant course was complicated with immune thrombocytopenic purpura (ITP) secondary to infection and was treated successfully with weekly rituximab and later with eltrombopag. His blood counts normalized, and he remained in remission with regular follow-ups. The patient received Pfizer-BioNTech COVID-19 vaccination series on March 12, 2021, and April 2, 2021. His follow-up labs in late March 2021 showed transaminitis, with aspartate transaminase (AST) of 221 U/L (normal: 5-45 U/L) and alanine transaminase (ALT) of 472 U/L (normal: 5-60 U/L). No alcohol or other substance ingestion was reported. Hepatitis A, B, and C serologies were negative. Serum Epstein-Barr virus (EBV) and cytomegalovirus (CMV) PCR assays were also negative. The patient complained of oral white plaques, dry mouth, and dry eyes. He was diagnosed with GVHD grade 2 and was started on tacrolimus 1 mg in the morning and 0.5 mg in the evening along with artificial tear, dexamethasone oral rinse, and nystatin oral solution, with improvement in his symptoms and liver function tests. The latest results from September 2021 showed AST of 43 U/L and ALT of 60 U/L. He is currently maintained on tacrolimus 0.5 mg twice daily and sorafenib 200 mg twice a day. The patient was also being followed for right kidney lesions. He underwent right partial nephrectomy and histopathologic evaluation showed renal cell carcinoma. He is being followed up with imaging surveillance.

Case 2

The second case is of a 61-year-old female with a history of treated thymoma in 2010 who presented initially in May 2018 with leukocytosis and was diagnosed with Philadelphia chromosome (+) B-cell acute lymphoblastic leukemia (ALL) without central nervous system involvement. The patient received induction with HyperCVAD (cyclophosphamide, vincristine, doxorubicin, dexamethasone) plus dasatinib. After achieving complete remission without minimal residual disease, she was referred for AHSCT, which was done in November 2018. The patient did well, and her follow-up visits were unremarkable. She received her Pfizer-BioNTech COVID-19 vaccination series on February 25, 2021, and March 8, 2021. In April 2021, the patient had oral symptoms in the form of white plaquelike lesions, and her AST and ALT acutely increased, peaking to 500 U/L and 641 U/L, respectively. No alcohol or other substance ingestion was reported. Serum EBV was negative. Hepatitis A, B, and C serologies were also negative. A liver biopsy was obtained and was reported as non-specific chronic active hepatitis that may be immune mediated (e.g.: drug-induced) and/or autoimmune with immunohistochemical stains for adenovirus and CMV being negative. She was started on oral prednisone 60 mg daily, with immediate improvement of liver function tests and oral symptoms. Her liver function test rose again after being taken off the steroid taper in July 2021. She was restarted on a moderate dose of prednisone 20 mg daily and is currently undergoing a very slow taper. Her latest AST and ALT results from September 2021 were 23 U/L and 27 U/L, respectively.

## Discussion

The estimated annual number of AHSCT increased gradually over the past years. It is estimated that the number of recipients surpassed 8,000 a year since 2013, reaching 9,498 estimated transplants in the year 2019 [8]. Among unrelated donor AHSCT, the mortality related to GVHD is 16% within 100 days of transplant and 11% after 100 days [8]. The current understanding of biological and molecular events that occur in GVHD is based mostly on murine studies, and the earliest pathophysiological events that occur in GVHD include infiltration of the tissue with innate myeloid cells such as neutrophils and monocytes [1]. Inflammation is triggered by the activation of T cells by two broad categories of molecules: sterile damage-associated molecular pattern (DAMP) molecules and pathogen-associated molecular pattern (PAMP) molecules. DAMPs are molecules that release into the extracellular space at the site of tissue damage, causing immune activation (e.g., ATP, uric acid). PAMPs are released by various bacteria, fungi, and viruses, resulting in priming of T cells and immune activation [1].

As described previously, vaccine administration is important after recovery of the immune system following AHSCT. Vaccines are formed from antigenic components of pathogens aiming to activate the immune system and form immunoglobulin against that pathogen. On November 11, 2020, the Food and Drug Administration (FDA) issued an Emergency Use Authorization (EUA) to permit the use of Pfizer-BioNTech COVID-19 vaccine to prevent COVID-19. The most common local side effect of Pfizer-BioNTech COVID-19 vaccine was pain at injection site [9]. On the other hand, fatigue and headaches were reported to be the highest systemic side effects [9]. Moreover, there have been reports of more severe reactions such as anaphylaxis [10]. The literature is lacking regarding the relation between vaccines in general and the development or worsening of GVHD in recipients of AHSCT. However, a recent study retrospectively examining the safety and efficacy of the SARS-CoV-2 EUA vaccines in 113 AHSCT recipients after receiving at least one of EUA vaccines (including the Pfizer and Moderna vaccines) reported that 40% of patients who received the EUA vaccines had chronic GVHD at the time of vaccination and that worsening of chronic GVHD occurred in 3.5% of the patients [11]. The study also shows that 9.7% of the patients developed new chronic GVHD after vaccination, but it does not specify the percentages for individual vaccines [11]. All of the patients who developed new or had worsening GVHD after vaccination were above the age of 40, and the onset occurred within 60 days from vaccination [11].

Our two cases were doing well without clinical or laboratory evidence of GVHD until they received doses of the Pfizer-BioNTech COVID-19 vaccine. The main differential diagnoses considered were infectious, drug-related reactions, GVHD, ischemic hepatitis, alcoholic hepatitis, paracetamol-induced hepatitis, or other primary autoimmune disorders. Both patients did not use alcohol, developed hypotension, or reported excess paracetamol ingestion, which usually present more acutely and cause significant elevations in liver function. They were screened for viral hepatitis, EBV, and CVM infections. Additionally, neither patient was started on any new medications prior to their new findings. Both patients developed oral symptoms with a spike in liver function test within one month of the first or second dose of the vaccine. GVHD diagnoses were supported by classical appearance of the oral lesions and prompt response to immunosuppressive therapy. This is similar to the pattern reported by Ali et al. [11] in which the onset of GVHD occurred within 30 days of vaccination in most patients. The molecular components of these vaccines may serve as PAMPs triggering the immune response, but since it is not seen with every patient receiving the vaccine, we hypothesize that there may be other unique signaling pathways involved or antigen mismatching resulting in such an event.

## Conclusions

SARS-CoV-2 vaccination is recommended in immunocompromised patients including recipients of AHSCT. The risk of GVHD may increase after vaccine administration in this group of patients and thus require closer monitoring after administration. A larger scale study is required to prove this association and underlying molecular mechanism.
